# Running wild: Forest running enhances performance, enjoyment, mood, and future exercise intentions

**DOI:** 10.1093/emph/eoag018

**Published:** 2026-08-27

**Authors:** Matthew J Putland, Stephen J Bailey, Bryna C R Chrismas, Stacy Clemes, Morgan Ewers, Yu-Ling Ho, Eloise Jones, Adelina Lintuluoto, Kirsten McEwan, Riddhi Naik, Hanna L Rahimi, Hongbin Xiang, Erik P Willems, Colin N Shaw, Daniel P Longman

**Affiliations:** School of Sport, Exercise and Health Sciences, Loughborough University, Loughborough, UK; School of Sport, Exercise and Health Sciences, Loughborough University, Loughborough, UK; School of Sport, Exercise and Health Sciences, Loughborough University, Loughborough, UK; School of Sport, Exercise and Health Sciences, Loughborough University, Loughborough, UK; School of Design and Creative Arts, Loughborough University, Loughborough, UK; School of Sport, Exercise and Health Sciences, Loughborough University, Loughborough, UK; School of Sport, Exercise and Health Sciences, Loughborough University, Loughborough, UK; CERN, Geneva, Switzerland; College of Health, Psychology and Social Care, University of Derby, Derby, UK; School of Sport, Exercise and Health Sciences, Loughborough University, Loughborough, UK; School of Sport, Exercise and Health Sciences, Loughborough University, Loughborough, UK; School of Sport, Exercise and Health Sciences, Loughborough University, Loughborough, UK; Department of Evolutionary Anthropology, University of Zurich, Zurich, Switzerland; Department of Evolutionary Anthropology, University of Zurich, Zurich, Switzerland; School of Sport, Exercise and Health Sciences, Loughborough University, Loughborough, UK

**Keywords:** environmental mismatch hypothesis, green exercise, exercise performance, public health, physical activity, physical inactivity

## Abstract

**Background and objectives:**

Physical inactivity remains widespread despite the well-established physical and mental health benefits of regular physical activity (PA). There is an urgent need for strategies that increase both uptake and long-term adherence. The *Environmental Mismatch Hypothesis* proposes that natural environments may increase both engagement and performance of PA.

**Methodology:**

Forty-seven healthy adults (16 females) completed a randomized, counterbalanced crossover trial. Participants undertook a 30-min self-paced run in both a forest and an urban environment at a prescribed exertion level (rate of perceived exertion = 4/10). Performance (distance run, heart rate) and markers of intention to perform future PA (enjoyment, mood, short-, and long-term intention) were assessed.

**Results:**

Participants covered significantly greater distance in the forest condition (*P* = .036, Cohen’s *dz* = 0.2) with heart rate and rate of perceived exertion comparable to the urban setting. Forest-based exercise also elicited significantly greater enjoyment (*P* < .001, *dz* = 1.3), improved post-exercise mood (*P* < .001, *dz* = 0.7) and far greater intention to engage in future PA (short-term *dz* = 2.1; long-term *dz* = 2.0; both *P* < .001).

**Conclusions and implications:**

Forest-based running was associated with improved performance, enjoyment, mood, and motivation for future PA, relative to urban running, without an increase in physiological strain. These findings reveal that natural environments offer a promising and scalable approach to addressing physical inactivity and improving population health. Further research is required to determine the environmental drivers, the feasibility, accessibility, and long-term effects across diverse and inactive populations.

## BACKGROUND AND OBJECTIVES

### Physical inactivity: A global public health crisis

Physical inactivity is a pervasive and pressing public health challenge worldwide. Despite the well-established physical and mental health benefits of regular physical activity (PA) [1], around one-third of adults—approximately 1.8 billion people globally—fail to meet the World Health Organization’s (WHO) recommendation of 150 min of moderate-intensity or 75-min of vigorous-intensity PA per week [2]. Even amongst adults who do engage in exercise, adherence remains low, with an estimated 63% of new fitness club members discontinuing activity within 3 months and ˂4% maintaining consistent participation beyond 1 year [3].

Globally, physical inactivity imposes a substantial health and economic burden. Insufficient PA is a major risk factor for a litany of non-communicable diseases (NCDs), including cardiovascular and cardiometabolic conditions, musculoskeletal disorders, impaired immune, and cognitive function, poor mental health, expedited ageing, obesity, and elevated risk of several cancers [1]. A 25% global increase in PA would prevent an estimated 1.3 million deaths annually [4] and greatly reduce the $27 billion in yearly worldwide healthcare costs attributable to inactivity [2]. Developing strategies that increase the uptake, performance, and long-term adherence of PA is therefore a public health priority.

### Nature: A promising solution to physical inactivity

Numerous initiatives have sought to enhance PA enjoyment through technology, gamification, and social interaction [5–7]. However, the influence of the physical environment is often overlooked. Emerging evidence indicates that PA performed in natural settings elicits greater enjoyment and more positive mood changes than activity undertaken in urban or indoor environments [8, 9]. This is important because enjoyment and mood are strong predictors of both short- and long-term exercise adherence [10, 11]. Beyond adherence, affective responses may also shape how physical effort is perceived. Experimental and theoretical work suggests that more positive affect can increase tolerance for higher exercise intensities without a corresponding increase in perceived exertion [12]. Given that natural environments are associated with more positive affective responses to exercise [13–15], such settings may enable individuals to sustain greater physical workloads at a similar perceived effort. This is particularly relevant from a public health perspective, as higher-intensity physical activity confers additional cardiometabolic and health benefits compared with lower-intensity activity [16].

Despite providing some theoretical and empirical insights, the *Green Exercise* literature, which investigates the benefits of exercising in natural environments, remains underdeveloped, and inconclusive. Systematic reviews and meta-analyses highlight notable methodological limitations, including inconsistencies in study design, inadequate sample sizes, and insufficient control of key contextual variables such as weather [8, 17, 18]. These issues lead to concerns about the reliability and reproducibility of some previous findings, underscoring the need for more rigorous experimentation to assess the efficacy of nature-based interventions to improve PA.

An emerging evolutionary perspective predicts that natural environments may also enhance physical performance. The *Environmental Mismatch Hypothesis* [19]*,* which extends the broader concept of evolutionary mismatch [20–25] to consider the implications of environmental industrialization on human evolutionary fitness, posits that human biology and function may be impaired by industrialized environments and enhanced by natural environments. Throughout *Homo sapiens’* evolutionary journey, varied natural environments defined the ecological parameters within which selection shaped our biology [26–28]. However, our habitat has undergone a rapid and profound transformation during the 200–300 years since the onset of the Industrial Revolution. Whilst ancestral environments were dominated by naturally occurring features and materials, contemporary environments are largely human made, incorporate fewer natural features, and often impose novel stressors such as sound, light, and chemical pollution. This rapid and profound environmental transition appears to adversely affect human biology, with previous work linking environmental industrialization with dysregulation of stress response systems, reductions in general health, and impairment of key biological functions that underpin evolutionary fitness [19, 29–38].

The *Environmental Mismatch Hypothesis* considers adaptation as the process by which phenotype is adjusted to optimize fitness within a specific environment [39]. Humans possess a hierarchical adaptive system that responds to environmental stressors to preserve homeostasis, function, and evolutionary fitness. As a species, we respond to environmental stressors through various processes, each operating on different timescales [40]. Immediate responses to environmental stressors include behavioural adaptation (e.g. seeking shade in response to heat) and physiological adaptation (e.g. sweating), which function as rapid, short-term adjustments. Over longer timescales, adaptation via phenotypic plasticity (e.g. musculoskeletal adaptation to novel physical activities) or developmental plasticity (e.g. stature influenced by childhood nutrition) enables individuals to adjust to environmental challenges within a lifetime [41, 42]. Across generations, epigenetic inheritance modifies patterns of gene expression in response to parental environments (e.g. reduced metabolic rate in offspring whose parents experienced a nutritionally deficient environmental deficit [43]).

Each of these adaptive processes helps to buffer the effects of environmental stressors on human biology without requiring underlying genetic change [44]. This buffering facilitates homeostasis—the self-regulating processes that maintain internal physiological stability despite external perturbation—thereby preserving biological function and evolutionary fitness. However, if this adaptive buffering fails to maintain homeostasis and function, adaptation via natural selection drives long-term evolutionary change by favouring heritable traits that enhance survival and reproduction, leading to population-level adaptation [45].

The *Environmental Mismatch Hypothesis* applies this hierarchical framework to contemporary industrialization. Critically, the faster-acting mechanisms (behavioural and physiological adaptation, phenotypic and developmental plasticity, and epigenetic inheritance) buffer environmental stress without requiring genetic change. The *Environmental Mismatch* framework predicts that contemporary industrialized environments may exceed the adaptive buffering capacity provided by these mechanisms, resulting in measurable impairment of function. This hypothesis does not require evidence of natural selection; it predicts that challenges exist at the proximate level, through specific environmental stressors (e.g. air pollution, noise, artificial light) that contemporary humans encounter, almost continuously, in more industrialized environments.

Physical function, the capacity to perform tasks requiring endurance and/or strength, is a fundamental determinant of survival, reproduction and, therefore, evolutionary fitness. Across the hominin lineage, selective pressures appear to have favoured endurance capacity in particular. Unlike other primates, *Homo sapiens* evolved a unique capacity for sustained endurance activity, supported by a suite of anatomical and physiological adaptations that enhanced locomotor efficiency, strength, stabilization, and thermoregulation [46–48]. Throughout human evolution, endurance capacity was critical for survival and reproduction. Behaviours such as hunting, scavenging, and seasonal mobility, which were essential for acquiring food and water and navigating changing climatic conditions, demanded prolonged physical effort over long distances [46]. Hunting, for example, provided access to calorie-dense animal foods critical for survival and provisioning, with endurance running emerging as a central component of hunting success in some human groups [49, 50]. Endurance capacity may also have been reinforced through social and reproductive mechanisms [51], as physically demanding activities such as hunting served as reliable signals of desirable traits (including endurance, strength, intelligence, and altruism) enhancing social status, mate attraction and reproductive success [52–56]. Thus, endurance capacity emerged as a key determinant of both survival and reproductive success.

Consistent with the *Environmental Mismatch Hypothesis*, our group recently reported that acute exposure to industrialized environments impaired subsequent laboratory-based cycling endurance performance by ~7.5% compared to forest exposure, providing experimental support for the prediction that physical function is optimized in natural settings [57]. Given that physical function continues to strongly predict health outcomes in contemporary populations and remains a measurable marker of evolutionary fitness [46, 47, 55, 58], it is hypothesized that PA performance will be optimized in environments that more closely resemble those to which human physiology is primarily adapted—namely, natural rather than urbanized settings [57].

To evaluate whether industrialization impacts physical function as predicted by the *Environmental Mismatch Hypothesis*, we conducted a randomized, counterbalanced crossover experiment in which participants performed moderate-intensity exercise indicative of real-world PA in a forest and an urban environment. Running performance (distance covered), heart rate, perceived effort and markers of intention to perform future PA (enjoyment, mood and short-, and long-term intention) were assessed.

## METHODOLOGY

### Participants and ethics

Healthy adults aged 18–31 years were recruited from Leicestershire, UK (Table 1). Inclusion criteria included body mass index (BMI) < 30 kg/m^2^ and blood pressure < 140/90 mmHg. Exclusion criteria included diagnosed mood disorders, contraindications to exercise, amenorrhoea, smoking, regular alcohol use (> 14 units per week), pregnancy or nursing. Forty-seven participants (31 males, 16 females: 38 from East Asia, 8 white European and 1 undisclosed) completed the study. Ethical approval was granted by the Loughborough University Ethics Review Human Participants Sub-Committee (Application No: 2024-17721-18835) and all participants provided written informed consent.

**Table 1:** Participant descriptive statistics.

	Male (*n* = 31)		Female (*n* = 16)	
	Mean (*SD*)	Range	Mean (*SD*)	Range
Age (Y)	24.4 (2.8)	18–30	25.5 (3.4)	20–31
Height (m)	1.78 (0.07)	1.64–1.93	1.66 (0.08)	1.50–1.80
Mass (kg)	72.7 (10.2)	52.8–92.2	61.2 (8.9)	42.8–76.6
BMI (kg/m^2^)	23.2 (2.6)	17.5–27.4	22.0 (2.4)	19.0–28.5

### Study design

All participants completed the randomized counterbalanced crossover trial with trial order balanced within each sex.

### Familiarization session

During an initial familiarization session at Loughborough University, participants were instructed on the modified Borg category-ratio (CR-10) rate of perceived exertion (RPE) scale [59]. CR-10 is an extensively validated category-ratio scale [60], ranging from 0 (rest) to 10 (maximal effort). Participants were informed that they needed to maintain a moderate-intensity effort (RPE = 4/10), which reflects a sustainable intensity described as ‘somewhat hard’—comfortable but requiring some effort, often when speech begins to become broken. This intensity was chosen to optimize application to public health, as it matches the minimum exercise intensity required for long-term health benefits [61] whilst accommodating individual fitness variation. Participants practised maintaining this exertion level whilst running or walking on a ~ 200 m marked outdoor circuit. Throughout, participants wore a POLAR Unite 10 watch and H10 chest strap (Polar Electro Oy, Kempele, Finland) to familiarize themselves with the monitoring devices used during testing.

### Pre-exercise protocol

Participants abstained from PA 24-h before testing, from alcohol and/or other intoxicating substances the evening prior to testing and from caffeine on the day of testing. They were instructed to wear suitable active clothing and footwear for both sessions. Upon arrival at 08:30 at Loughborough University’s National Centre for Sport and Exercise Medicine (NCSEM), participants sat quietly for 10 min ahead of baseline assessments. Phones and smartwatches were silenced and stored throughout the entire session.

Baseline measures included the following.

Blood pressure (BP) and heart rate (HR): Measured using a sphygmomanometer (M6, Omron, Kyoto, Japan) on the non-dominant upper arm whilst the participant was sitting upright. Three separate measurements of BP and HR were taken at 30-s intervals. The mean of the second and third assessments were calculated and used.

Mood: Assessed via the abbreviated 40-item Profile of Mood States (POMS) questionnaire, a validated psychological tool for evaluating transient affective states [62]. The questionnaire captures seven dimensions—tension, depression, fatigue, vigour, confusion, anger, and esteem-related affect—which together yield a composite mood score (‘total mood disturbance’).

Habitual physical activity: Measured using the 16-item Global Physical Activity Questionnaire (GPAQ), developed by the World Health Organization (WHO) [63]**.** Weekly PA levels were calculated in MET minutes per week. Most participants met WHO guidelines [64] of moderate PA (29/31 males, 15/16 females), and a majority also met the higher threshold for additional health benefits (21/31 males, 13/16 females). Measured once at baseline pre-exercise (see Supplementary Information 1 for further details).

Participants were then driven (∼9 min) to either Bishop Meadow Industrial Estate (urban condition) or The Outwoods ancient woodland, Charnwood (forest condition), both in Loughborough, Leicestershire, UK (Supplementary Information 2). Upon arrival, participants walked ∼5 min to their unique starting positions along the exercise circuit. Prior to the onset of exercise, resting heart rate was recorded for each participant and running was initiated ˂10 min after arriving at the site.

### Exercise protocol

Participants completed a 30-min self-paced run/walk at RPE = 4/10 in mixed-sex groups of four. To minimize peer influence, participants were spaced around the circuit so as not to begin in sight of one another—though overtaking naturally occurred whilst running. Group composition varied randomly between sessions.

The two circuits were purposefully chosen to be as similar in length (urban: 633 m; forest: 630 m), topography, and substrate type as possible. The forest circuit had a slightly higher elevation change per lap (17.7 m vs 0.7 m per lap) and more uneven terrain, whereas the urban circuit had a uniform, less undulating concrete surface (Fig. 1). Measurements taken during the run included the following.

**Figure 1:**
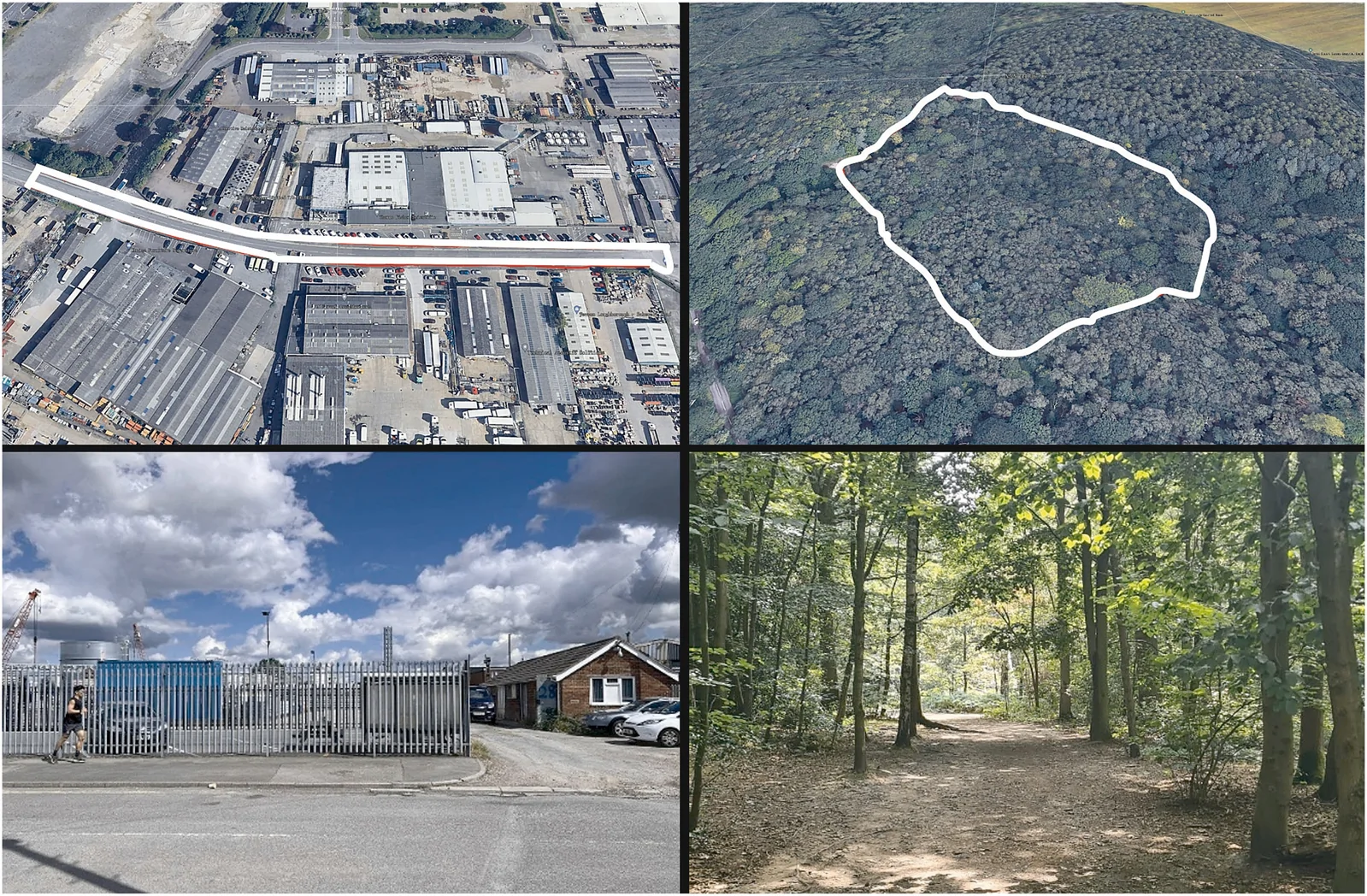
Visualization of the two exercise circuits. Satellite views (urban: upper left, forest: upper right), and photographs (urban: bottom left, forest: bottom right) of the two exercise circuits.

Distance covered: Measured using a lap counter and metre measuring wheel (Qingdao Qingqing Hardware Tools Co., Ltd, China). Participants were unaware of distance tracking.

Heart rate: Continuously recorded during exercise with a POLAR Unite 10 watch paired with a Polar H10 chest strap [65].

### Post-exercise protocol

Immediately post-exercise, participants reported the following.

Perceived exertion: Borg CR-10 Scale [59].

Enjoyment: Measured using the validated 18-item Physical Activity Enjoyment Scale (PACES) [66]**.**

Mood: Assessed via the validated abbreviated 40-item Profile of Mood States (POMS) questionnaire [62].

Future PA intention: Participants rated their intention to repeat the exercise in response to two questions: (i) ‘If you had the opportunity to do the same exercise in this location next week, how likely are you to do that?’ and (ii) ‘If you had the opportunity to do the same exercise in this location again, could you see yourself doing this regularly over the next 12 months?’ Responses were recorded on a 10 cm visual analogue scale ranging from ‘I definitely wouldn’t’ to ‘I definitely would’. This approach has been widely adopted in acute exercise research to assess behavioural intentions following single exercise bouts and provides a clear index of short- and long-term exercise motivation [67–69]**.**

The second intervention session occurred 4 weeks after the first, which provided an adequate washout period [70]. Female participants completed both sessions during days 1–10 of their menstrual cycle (self-reported) on self-selected days during which they were not experiencing menstrual-related symptoms, to minimize the effect of cycle-related variation on outcome variables [71].

### Environmental conditions

Weather and temperature were recorded using the official Met Office weather app and ground conditions (i.e. non-slippery vs slippery) were recorded during each session via investigator inspection.

### Statistical analyses

All analyses were conducted in SPSS v29, with statistical significance set at 0.05.

Fisher’s Exact Test compared the frequency of rainy or slippery conditions across environments and paired-samples t-tests assessed temperature differences and resting heart rate between environments.

Primary analyses used Gaussian linear mixed-effects models to evaluate the effect of environment on outcome variables. For most outcomes, models include fixed effects of environment, sex, environment x sex and trial order, with participant ID and group ID to account for repeated measures and clustering within exercise groups (i.e. the participants who exercised together in each session). For mood, which was measured both pre- and post-run, models additionally included time (pre−/post-) and the environment x time interaction. All models specified a normal probability distribution with an identity link function. Estimated marginal means (EMMs) were calculated to obtain covariate-adjusted condition mean-values.

Model residuals were assessed for normality (Shapiro–Wilk tests, histograms and Q-Q plots), homoscedasticity (residual vs. predicted scatterplots) and outliers (residual boxplots). Logarithmic transformations were applied where assumptions were violated.

Effect sizes (Cohen’s *dz*) were estimated from EMMs and their standard errors. Condition-specific *SD*s were estimated by multiplying the *SE* of the EMM by √*n*, and *dz* was computed as the difference between adjusted means of the two conditions divided by the pooled *SD*.

## RESULTS

### Baseline measures

There were no significant baseline differences in mood or physiological stress (i.e. heart rate and blood pressure) between environments measured at the NCSEM laboratory.

### Weather conditions

Temperature (forest: *M* = 20.5°C, *SD* = 4.0°C; urban: *M* = 19.6°C, *SD* = 3.8°C; mean difference = −0.9°C, 95% CI [−2.5, 0.6], *P* = .243) and number of rainy test sessions (forest: 11/47 days; urban: 14/47 days; Fisher’s Exact Test, *P* = .483) did not differ between sites. The ground was slippery more frequently in the forest (slippery due to mud for 15/47 participants) than in the urban setting (slippery due to a wet pavement for just 1/47 participants); Fisher’s Exact Test, *P* < .001.

Model statistics for these analyses are presented below and expanded upon in Supplementary Information 3. Table 2 presents estimated marginal means (EMMs), *P*-values and effect sizes.

**Table 2:** Summary of key outcome measures following exposure to forest and urban environments. Values are estimated marginal means (EMMs ± *SE*) derived from linear mixed effects models. For short- and long-term intention, enjoyment, running distance, and rating of perceived exertion, models included environment and trial order as fixed effects and participant ID as a random effect. For mood, models additionally included time (pre−/post-run) and environment × time interactions. Reported *P*-values correspond to the fixed effect of environment (or environment × time where applicable). Effect sizes (Cohen’s *dz*) are shown for significant or near-significant effects. See Supplementary Information 4 for raw data means partitioned by sex.

Variable	Forest EMM ± *SE*	Urban EMM ± *SE*	Estimated difference (95% CI)	Environment/Environment × Time interaction *P*-value	Cohen’s *dz*
Running distance (m)	4118 ± 123.0	3936 ± 123.3	182 [13.0, 351.2]	0.036	0.2
Rating of perceived exertion	4.3 ± 0.1	4.4 ± 0.1	0.1 [−0.4, 0.2]	0.531	-
Heart rate (bpm)	157.7 ± 2.1	158.1 ± 2.1	−0.4 [−4.2, 3.4]	0.822	-
Short-term intention (%)	77.4 ± 3.2	30.6 ± 3.2	46.8 [37.7, 55.9]	<0.001	2.1
Long-term intention (%)	71.5 ± 3.6	21.0 ± 3.6	50.4 [40.7, 60.2]	<0.001	2.0
Enjoyment	98.7 ± 2.8	73.9 ± 2.8	24.8 [17.6, 32.17]	<0.001	1.3
Mood	89.8 ± 2.2 ➔80.3 ± 2.2	87.8 ± 2.2 ➔91.1 ± 2.2	Forest: −9.5 [−13.1, −5.9] Urban: 3.3 [−0.3, 7.0]	<0.001	0.7

### Markers of performance

#### Running distance

Participants ran significantly further in the forest compared with the urban condition (*F*(1,20) = 5.1, *P* = .036; Table 2, Fig. 2). The effect size, however, was small (*dz* = 0.2).

**Figure 2:**
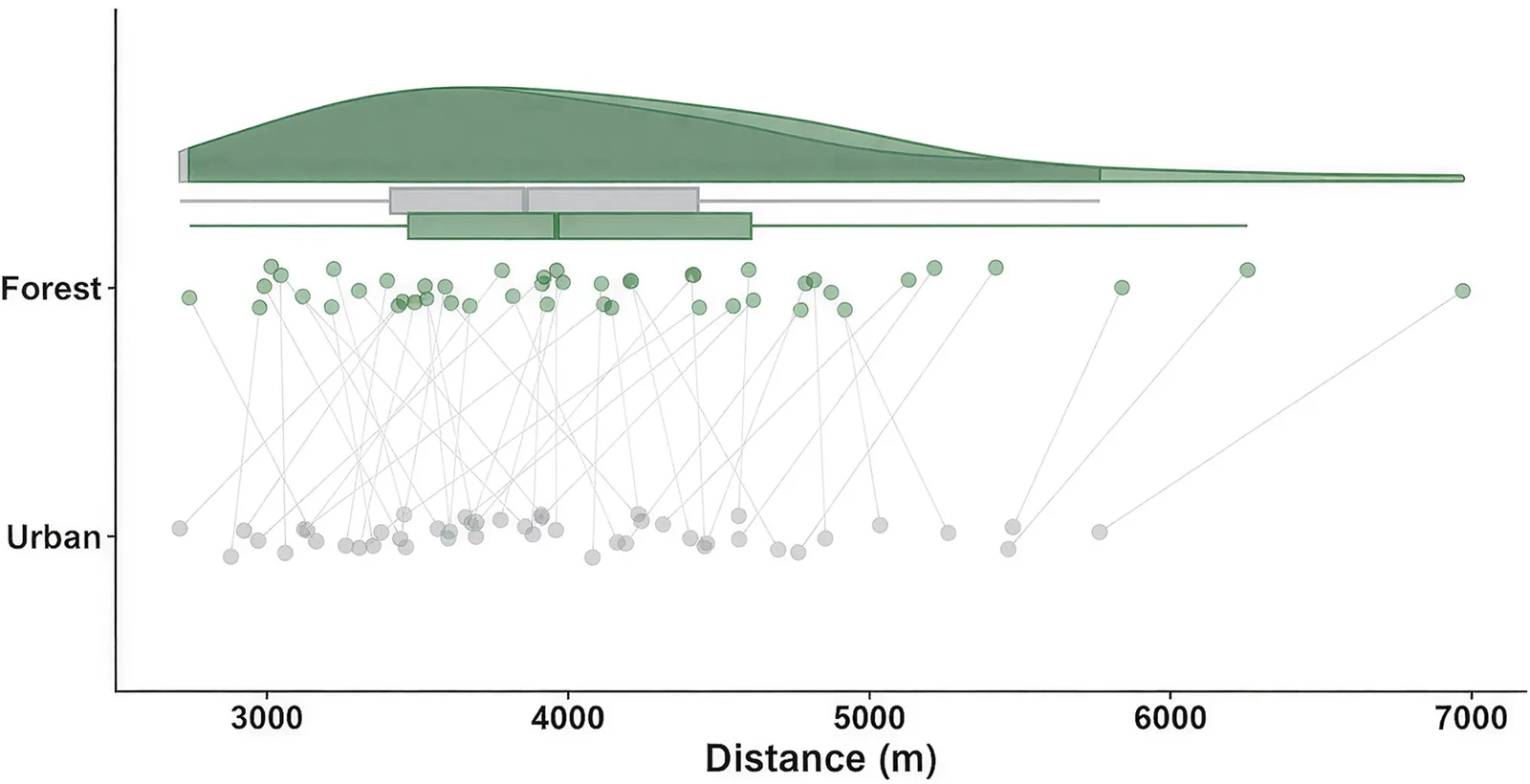
Visualization of exercise performance in the forest (green) compared to the urban control (grey) environment. Distance completed was significantly greater when exercising in the forest compared to the urban control environment. Raincloud plot code from [72].

#### Perceived exertion (RPE)

There was no significant effect of environment on perceived exertion during the run (*F*(1,91) = 0.4, *P* = .531, Table 2).

#### Heart rate

Resting heart rate, measured immediately prior to exercise, did not differ significantly between the forest (M = 86.1 ± 20.5 bpm) and urban (M = 80.7 ± 12.0 bpm) environments, t(37) = 1.65, *P* = .108. There was no significant effect of environment on mean heart rate during the run (*F*(1,73) = 0.051, *P* = .822, Table 2). Similarly, there was no difference in within-person variation in heart rate during exercise between the forest (SD = 12.6 ± 2.8 bpm) and urban (SD = 12.2 ± 2.1 bpm) conditions, t(36) = 1.02, *P* = .315.

### Markers of intention to perform future physical activity

#### Short- and long-term intentions

Participants reported significantly greater short- and long-term intentions to exercise following the forest session compared with the urban session (short-term: *F*(1,19) = 115.5, *P* < .001; long-term: *F*(1,91) = 108.0, *P* < .001; Table 2, Fig. 3). Effect sizes were very large (short-term *dz* = 2.1; long-term *dz* = 2.0) and the two measures were highly correlated (*r* = 0.871, *P* < .001). A marginal environment × sex interaction was observed for both outcomes (short-term: *P* = .053; long-term: *P* = .063), suggesting that the increase in intention to perform future PA following the forest versus urban session was more pronounced in female than male participants.

**Figure 3:**
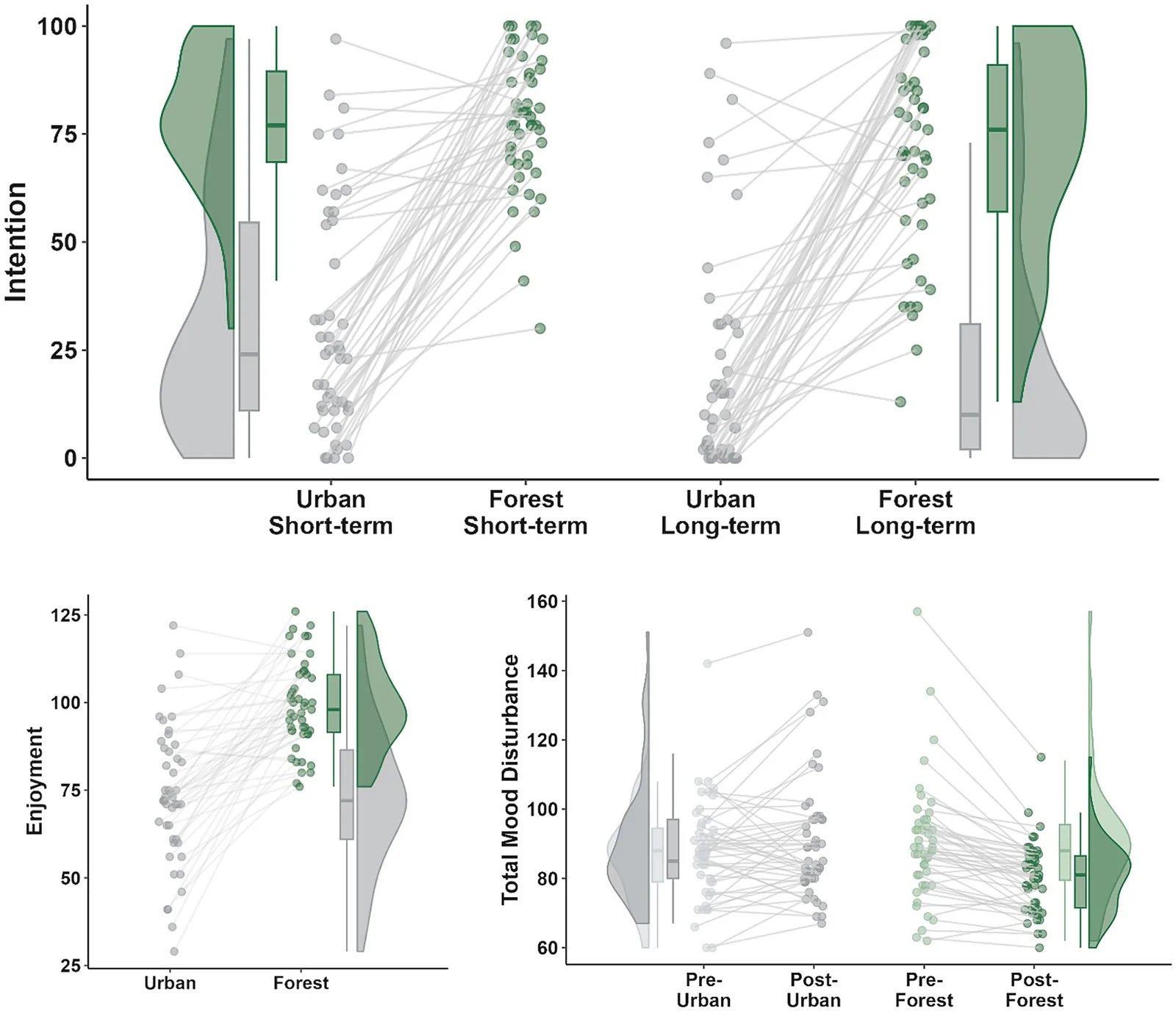
Visualization of short- and long-term intention to engage in future physical activity (top row), enjoyment of physical activity (bottom left row) and mood, as measured by total mood disturbance (TMD) (bottom right row). For TMD, a lower score indicates better mood. Short- and long-term intention to exercise, enjoyment, and improvement in mood were significantly greater following exercise in the forest compared to the urban control environment [72]. Forest condition for intention and enjoyment is represented by dark green and urban control by dark grey. For mood, forest condition is represented by light green (pre) and dark green (post); urban control by light grey (pre) and dark grey (post).

#### Enjoyment

Participants reported significantly greater enjoyment of exercise in the forest than in the urban environment (*F*(1,33) = 48.8, *P* < .001; Table 2, Fig. 3), with a large effect size (*dz* = 1.3).

#### Mood

A significant environment x time interaction was found for total mood disturbance (*F*(1,182) = 24.594, *P* < .001), whereby mood improved more after running in the forest than after running in the urban location (Table 2, Fig. 3). The effect size was 0.7, indicating a moderate effect. Whilst pre-session mood did not differ between environments, post-run mood was significantly better following the forest session compared against the urban session.

Unless stated otherwise, for each of the variables assessed above, additional models showed that the effect of the forest environment on the outcome was not significantly influenced by participant sex, age, BMI, baseline running performance (in the urban environment), weekly physical activity, preference for forest vs urban environments or childhood environment (farm vs village vs suburb vs city centre).

## DISCUSSION

### Physical performance improves in the forest

We hypothesized that physical performance would be enhanced in the forest environment. This hypothesis was supported, as participants covered significantly greater distances during the 30-min walk/run in the forest (+182 m, a 4.6% increase), despite more challenging terrain and similar perceived (RPE) and physiological (mean heart rate) effort. The forest course included elevation change (17.7 m per lap), somewhat uneven surfaces and occasionally slippery terrain. Running on uneven natural surfaces requires greater metabolic energy expenditure than smooth surfaces [73, 74] and the energetic cost of uphill running typically exceeds savings from matched downhill sections [75, 76], likely due to an increased demand for neuromuscular control and core stability [77]. In contrast, the urban route was flat (<1 m elevation gain per lap) and paved, offering firm, consistent surfaces that reduce energy loss and facilitate efficient forward propulsion [74, 78]. Thus, all else being equal, the forest course used in this study should have produced a slower pace. The finding that performance was nonetheless superior in the forest suggests that the influence of environment outweighed the potential terrain-related biomechanical challenges. If the terrain had been identical, it may have been that the distance covered in the natural environment would have been even greater.

Whilst the *Environmental Mismatch Hypothesis* [19] provides a potential ultimate explanation—positing that environmental industrialization imposes novel challenges that our nature-adapted biology is ill-equipped to cope with—several *proximate* mechanisms may contribute to the observed performance differences. For example, air pollutants such as fine and ultrafine particulate matter, nitrogen dioxide, and ozone can impair pulmonary and cardiovascular function and reduce exercise capacity [79, 80]. These effects are seen in slower 5 km and marathon times and lower V̇O₂_max_ across diverse populations [81–86]. Publicly available emissions data from the 2023 UK National Atmospheric Emissions Inventory suggest that the urban location used in this study may have featured markedly higher relative PM2.5 (particulate matter <2.5 μm) air pollution levels (1.16 tonnes/km^2^) than the forest site (0.04 tonnes/km^2^) [87]—approximately a 30-fold difference in emissions density. Separately, the ultrafine fraction of particulate matter (<0.1 μm) can penetrate deep into the lungs, impact the brain via the olfactory nerve and negatively affect both general health and physical performance [88]. However, air pollutant measurements were not collected along the running routes during the exercise sessions. Thus, any impact of air pollutants on running performance noted in this study remains speculative. Other mechanisms, such as psychological restoration, reduced mental fatigue, and beneficial sensory experiences in natural settings, could have played important roles. Future research that incorporates real-time air quality monitoring alongside measures of psychological state would help clarify the relative contributions of these mechanisms.

To our knowledge, this is the first robust experimental evidence of enhanced physical performance in natural environments. Whilst previous studies have reported more vigorous dancing in parks than in indoor dance studios [89] and faster running outdoors than on indoor treadmills [90], both suffer from methodological limitations that reduce interpretability. The work on the capacity to dance lacked description of the park environment, making it unclear just how natural or industrial it was. In the study of running capacity, differences may reflect the biomechanical and perceptual discrepancies between treadmill and overground running, making it difficult to isolate the role of the natural environment [91]. The present 4.6% (182 m) performance improvement represents an important proof of principle that natural environments can directly enhance physical capacity, even in the presence of biomechanical disadvantages.

Notably, in the present study, participants’ heart rate did not differ between conditions, despite running faster and encountering more challenging terrain in the forest condition. Thus, participants performed more physical work without greater physiological strain, which may be the result of mediation by psychological factors (i.e. mood). However, as heart rate is only a proxy for physiological strain, it cannot be stated definitively that similar heart rates between the two conditions reflect comparable energy expenditure in the forest environment. Future studies should directly measure energy expenditure during standardized PA in different environmental contexts to clarify whether observed performances are linked to differences in metabolic cost.

### Enjoyment, mood, and future intention improve in the forest

We also hypothesized that forest-based exercise would elicit greater enjoyment, mood (collectively referred to as positive affective responses) and future PA intentions compared against urban-based exercise. This hypothesis was confirmed for all three measures.

First, participants experienced significantly greater enjoyment during the forest session, despite running further, indicating that the natural environment enhanced the affective experience of PA [12]. Given that enjoyment is a key predictor of both short- and long-term adherence [11], these findings highlight the potential of nature-based exercise to promote sustainable behavioural change by enhancing intrinsic motivation to maintain regular PA.

Second, participants’ mood improved significantly after forest exercise, whereas it showed a non-significant trend towards worsening in the urban control condition. These results align with previous findings that nature-based exercise promotes more positive affective states than urban or indoor environments [8, 9]. According to Affective-Reflective Theory, positive affective responses can create strong, subconscious associations between activity and pleasure, increasing the likelihood of future participation and sustained engagement with PA [92].

Third, stated intentions to perform future PA in both the short-term and long-term were significantly greater after the forest exercise than the urban control. Although intentions do not always translate into action [93–95], meta-analytic evidence from the Theory of Planned Behaviour indicates they are strong predictors of future exercise behaviour [96, 97]**.** These effects were not only statistically significant but also large in magnitude, suggesting that natural environments can meaningfully strengthen motivation to be active.

Collectively, these findings suggest that natural settings can effectively foster the motivational and affective foundations necessary for long-term PA engagement.

### Public health implications

The observed results are both novel and potentially meaningful for public health, though replication in more diverse samples is needed. Participants covered greater distances within the same time frame, indicating that they performed more physical work and were able to exercise at a higher intensity without increasing their effort. This performance enhancement is noteworthy as it represents the first robust experimental evidence that natural environments can directly improve physical performance during ecologically valid exercise. Importantly, this performance gain was accompanied by substantially larger psychological benefits: markedly enhanced enjoyment, improved mood, and stronger intentions for future PA in the forest relative to the city. The combination of enhanced performance and large psychological effects is particularly promising from a public health perspective. Higher-intensity PA confers greater cardiovascular and metabolic health benefits than lower-intensity activity [16], whilst enjoyment and positive affect are primary drivers of long-term PA adherence [10, 11]. Natural environments may therefore offer dual benefits: enabling people to work harder physiologically whilst simultaneously making the experience more enjoyable and sustainable.

The benefits of the forest environment were not moderated by age, BMI, activity levels, baseline running performance, or environmental preference within our sample. However, replication in older, sedentary and more diverse populations is needed to establish broader generalizability. Nevertheless, a near-significant environment x sex interaction for future PA intentions suggests that women may experience greater motivational gains from nature-based exercise than men. This sex difference, however, may be partly attributable to the differences in running route configuration (generally circular vs. relatively out and back), which influenced how visible other participants were. Visibility during the city route may have influenced future PA in females, though this sex difference was not seen in other outcomes. Given the limited research into sex differences in biological responses to nature, this finding highlights a potential avenue for further investigation.

### Strengths, limitations, and conclusions

Strengths of this study include the simultaneous assessment of physiological and psychological outcomes from an ecologically valid exercise session, the relatively large sample and the rigorous experimental design. The 1-month washout period reduced order effects, and testing female participants within a controlled window of the menstrual cycle minimized hormonal influences [71]. Conducting the study within a 3-month window further reduced seasonal variability and differential environmental inputs. The consistency of effects across multiple outcome measures further strengthens confidence in the conclusions.

Limitations include minor differences in circuit terrain. Although efforts were made to match circuit distance and elevation, the forest circuit had slightly more challenging terrain (uneven surfaces and undulating topography). The findings are therefore conservative, as identical surface routes might have produced even more pronounced between-condition differences. The sample limits generalizability: participants were drawn from a narrow age range (18–31 years), were predominantly male (31 males, 16 females) and East Asian (81%), and were generally active (44 of 47 met WHO minimum PA guidelines [64]). Caution is therefore warranted when extrapolating to older adults, sedentary individuals, and more diverse populations. Lastly, the study measured intended rather than actual future PA. Although intention is a well-established predictor of behaviour [96], and mood and enjoyment are strong predictors of intention [98–100], intentions do not always translate into behaviour [93, 94].

These limitations point to several directions for future research. Replication in more diverse and sedentary populations is a priority, as is longitudinal work examining whether improvements in intention translate into sustained PA behaviour. To better understand the mechanisms underlying nature’s effects, studies should also combine physiological assessments with objective environmental measures. Finally, research should address the real-world sustainability of nature-based PA interventions, including disparities in access to safe natural spaces and variation in feasibility across seasons, work schedules, and socioeconomic groups.

In conclusion, this work supports the inclusion of evolutionary perspectives in the development of strategies to improve health in contemporary societies [24, 101]. By experimentally comparing ecologically valid and closely matched exercise sessions in forest and urban settings, this study provides novel evidence that natural environments can enhance both the experience and potential effectiveness of PA by:

1) Improving exercise performance at a given level of exertion.
2) Increasing enjoyment, improving mood and strengthening intentions to engage in future PA.

If these findings are replicated in more diverse and sedentary populations, natural environments offer a low-cost, scalable approach to supporting more sustainable and effective PA participation. These findings have implications for public health strategies aimed at promoting long-term PA engagement, particularly within urban populations.

## Supplementary Material

EMPH_supplementary_information_eoag018

## Data Availability

The data underlying this article are openly available in the Loughborough University Repository, at: Putland, Matthew (2026). Running wild: Forest running enhances performance, enjoyment, mood and future exercise intentions. Loughborough University. Dataset. https://doi.org/10.17028/rd.lboro.30904280.v2.

## References

[1] Warburton DER, Bredin SSD. Health benefits of physical activity: a systematic review of current systematic reviews. *Curr Opin Cardiol* 2017;32:541–56. 10.1097/HCO.0000000000000437 28708630

[2] WHO . World Health Organisation. 2024 [cited 2025 Jan 17]. Physical activity. https://www.who.int/news-room/fact-sheets/detail/physical-activity

[3] Sperandei S, Vieira MC, Reis AC. Adherence to physical activity in an unsupervised setting: explanatory variables for high attrition rates among fitness center members. *J Sci Med Sport* 2016;19:916–20. 10.1016/j.jsams.2015.12.522 26874647

[4] Lee IM, Shiroma EJ, Lobelo F et al. Effect of physical inactivity on major non-communicable diseases worldwide: an analysis of burden of disease and life expectancy. *Lancet* 2012;380:219–29. 10.1016/S0140-6736(12)61031-9 PMC3645500 22818936

[5] CDC . Strategies to Prevent Obesity and Other Chronic Diseases: The CDC Guide to Strategies to Increase Physical Activity in the Community. Atlanta: U.S. Department of Health and Human Services, 2011. https://www.cdc.gov/diabetes/news/media/pdfs/CDC-guide-strategies-increase-physical-activity.pdf.

[6] CDC . Centers for Disease Control and Prevention: Physical Activity. 2025 [cited 2025 Feb 15]. Strategies for Social Supports. https://www.cdc.gov/physical-activity/php/strategies/social-supports.html

[7] Edney S, Ryan JC, Olds T et al. User engagement and attrition in an app-based physical activity intervention: secondary analysis of a randomized controlled trial. *J Med Internet Res* 2019;21:e14645. 10.2196/14645 PMC6906621 31774402

[8] Lahart I, Darcy P, Gidlow C et al. The effects of green exercise on physical and mental wellbeing: a systematic review. *Int J Environ Res Public Health* 2019;16:8. 10.3390/ijerph16081352 PMC6518264 30991724

[9] Wicks C, Barton J, Orbell S et al. Psychological benefits of outdoor physical activity in natural versus urban environments: a systematic review and meta-analysis of experimental studies. *Appl Psychol Health Well-Being* 2022;14:1037–61. 10.1111/aphw.12353 PMC9544808 35259287

[10] Klompstra L, Deka P, Almenar L et al. Physical activity enjoyment, exercise motivation, and physical activity in patients with heart failure: a mediation analysis. *Clin Rehabil* 2022;36:1324–31. 10.1177/02692155221103696 PMC9420887 35678610

[11] Hagberg LA, Lindahl B, Nyberg L et al. Importance of enjoyment when promoting physical exercise. *Scand J Med Sci Sports* 2009;19:740–7. 10.1111/j.1600-0838.2008.00844.x 18694433

[12] Parfitt G, Hughes S. The exercise intensity–affect relationship: evidence and implications for exercise behavior. *J Exerc Sci Fit* 2009;7:S34–41. 10.1016/S1728-869X(09)60021-6

[13] Hansen MM, Jones R, Tocchini K. Shinrin-Yoku (Forest bathing) and nature therapy: a state-of-the-art review. *Int J Environ Res Public Health* 2017;14:8. 10.3390/ijerph14080851 PMC5580555 28788101

[14] Frumkin H, Bratman GN, Breslow SJ et al. Nature contact and human health: a research agenda. *Environ Health Perspect* 2017;125:075001. 10.1289/EHP1663 PMC5744722 28796634

[15] Bratman G, Anderson C, Berman M, Cochran B, de Vries S, Flanders J, Folke C, Frumkin H, Gross JJ, Hartig T, Kahn PH Jr, Kuo M, Lawler JJ, Levin PS, Lindahl T, Meyer-Lindenberg A, Mitchell R, Ouyang Z, Roe J, Scarlett L, Smith JR, van den Bosch M, Wheeler BW, White MP, Zheng H, Daily GC Nature and mental health: An ecosystem service perspective | Science Advances. 2019 [cited 2023 Dec 28]. 10.1126/sciadv.aax0903, 5 PMC6656547 31355340

[16] Garber CE, Blissmer B, Deschenes MR et al. Quantity and quality of exercise for developing and maintaining cardiorespiratory, musculoskeletal, and Neuromotor fitness in apparently healthy adults: guidance for prescribing exercise. Medicine & Science in Sports & Exercise 2011;43:1334–59. 10.1249/MSS.0b013e318213fefb 21694556

[17] Brito HS, Carraça EV, Palmeira AL et al. Benefits to performance and well-being of nature-based exercise: a critical systematic review and meta-analysis. *Environ Sci Technol* 2022;56:62–77. 10.1021/acs.est.1c05151 34919375

[18] Thompson Coon J, Boddy K, Stein K et al. Does participating in physical activity in outdoor natural environments have a greater effect on physical and mental wellbeing than physical activity indoors? A systematic review. *Environ Sci Technol* 2011;45:1761–72. 10.1021/es102947t 21291246

[19] Longman DP, Shaw CN. Homo sapiens, industrialisation and the environmental mismatch hypothesis. *Biol Rev* 2026;101:580–601. 10.1111/brv.70094 PMC12965856 41204658

[20] Lieberman DE . Is exercise really medicine? An evolutionary perspective. *Curr Sports Med Rep* 2015;14:313–9. 10.1249/JSR.0000000000000168 26166056

[21] Manus MB . Evolutionary mismatch. *Evol Med Public Health* 2018;2018:190–1. 10.1093/emph/eoy023 PMC6109377 30159142

[22] Lloyd E, Wilson DS, Sober E. Evolutionary mismatch and what to do about it: a basic tutorial. *Evol Appl* 2011;2:1–25.

[23] Gluckman P, Hanson M. Mismatch: Why our World No Longer Fits our Bodies. Oxford: OUP Oxford, 2006, 299.

[24] Nesse RM, Williams GC. Why we Get Sick: The New Science of Darwinian Medicine. New York: Vintage Books, Vintage books, 1996. 290.

[25] Lieberman D . The story of the human body : evolution, health, and disease. New York: Pantheon Books; 2014 [cited 2025 Feb 12]. 490 p. http://archive.org/details/storyofhumanbody0000lieb_e6t8 27875612

[26] Potts R . Environmental hypotheses of hominin evolution. *Am J Phys Anthropol* 1998;107:93–136. 10.1002/(SICI)1096-8644(1998)107:27+<93::AID-AJPA5>3.0.CO;2-X 9881524

[27] Potts R . Hominin evolution in settings of strong environmental variability. *Quat Sci Rev* 2013;73:1–13. 10.1016/j.quascirev.2013.04.003

[28] Potts R . Environmental and Behavioral evidence pertaining to the evolution of early homo. *Curr Anthropol* 2012;53:S299–317. 10.1086/667704

[29] Gluckman PD, Hanson MA, Low FM. Evolutionary and developmental mismatches are consequences of adaptive developmental plasticity in humans and have implications for later disease risk. *Philos Trans R Soc B Biol Sci* 2019;374:20180109. 10.1098/rstb.2018.0109 PMC6460082 30966891

[30] Gluckman PD, Low FM, Hanson MA. Anthropocene-related disease: the inevitable outcome of progressive niche modification? *Evol Med Public Health* 2020;2020:304–10. 10.1093/emph/eoaa042 PMC7723875 33324485

[31] Godfrey KM, Lillycrop KA, Burdge GC et al. Epigenetic mechanisms and the mismatch concept of the developmental origins of health and disease. *Pediatr Res* 2007;61:5R–10. 10.1203/pdr.0b013e318045bedb 17413851

[32] Gurven MD, Lieberman DE. WEIRD bodies: mismatch, medicine and missing diversity. *Evol Hum Behav* 2020;Beyond Weird;41:330–40. 10.1016/j.evolhumbehav.2020.04.001 PMC7584376 33100820

[33] Lea AJ, Clark AG, Dahl AW et al. Applying an evolutionary mismatch framework to understand disease susceptibility. *PLoS Biol* 2023;21:e3002311. 10.1371/journal.pbio.3002311 PMC10513379 37695771

[34] Low FM, Gluckman PD, Hanson MA. Niche modification, human cultural evolution and the Anthropocene. *Trends Ecol Evol* 2019;34:883–5. 10.1016/j.tree.2019.07.005 31422891

[35] McMichael AJ . Human Frontiers, Environments and Disease: Past Patterns, Uncertain Futures. Cambridge: Cambridge University Press, 2001, 430. 10.1017/CBO9781139106924

[36] Schell LM, Ulijaszek SJ. Urbanism, Health and Human Biology in Industrialised Countries. Cambridge: Cambridge University Press, 1999, 342. 10.1017/CBO9780511525698

[37] Dorsey AF . Urbanization and infectious disease. *Am J Hum Biol* 2025;37:e24197. 10.1002/ajhb.24197 39605171

[38] Schell LM . The biological challenge of urban environments: overview and introduction to the special issue on urbanism. *Am J Hum Biol* 2025;37:e70070. 10.1002/ajhb.70070 40444911

[39] Stock JT . Are humans still evolving? *EMBO Rep* 2008;9:S51–4. 10.1038/embor.2008.63 PMC3327538 18578026

[40] Stock J, Will M, Wells J. Evolutionary synthesis and distributed adaptation in the genus homo: phenotypic plasticity and Behavioral adaptability. *PaleoAnthropology.* 2023;2:205–33. 10.48738/2023.iss2.xxx

[41] Pigliucci M, Murren CJ, Schlichting CD. Phenotypic plasticity and evolution by genetic assimilation. *J Exp Biol* 2006;209:2362–7. 10.1242/jeb.02070 16731812

[42] Nettle D, Bateson M. Adaptive developmental plasticity: what is it, how can we recognize it and when can it evolve? *Proc R Soc B Biol Sci* 1812;282:20151005. 10.1098/rspb.2015.1005 PMC4528519 26203000

[43] Lacal I, Ventura R. Epigenetic inheritance: concepts, mechanisms and perspectives. *Front Mol Neurosci* 2018;11:292. 10.3389/fnmol.2018.00292 PMC6172332 30323739

[44] Stock J . Human evolution after the origin of our species: Bridging the gap between palaeoanthropology and bioarchaeology. In: Mitchell PD, Buckberry J (eds.), Proc 12th Annu Conf Br Assoc Biol Anthropol Osteoarchaeol (BABAO). Oxford: Archaeopress, 2012, 3–25. [cited 2026 Jul 29]. https://www.academia.edu/11873719/Human_Evolution_after_the_Origin_of_our_Species_Bridging_the_gap_between_Palaeoanthropology_and_Bioarchaeology

[45] Darwin C . On the origin of species by means of natural selection, or the preservation of favoured races in the struggle for life. 1859 [cited 2026 Jul 29]. http://archive.org/details/darwin-online_1859_Origin_F373 PMC5184128 30164232

[46] Bramble DM, Lieberman DE. Endurance running and the evolution of homo. *Nature.* 2004;432:345–52. 10.1038/nature03052 15549097

[47] Carrier DR, Kapoor AK, Kimura T et al. The energetic paradox of human running and hominid evolution. *Curr Anthropol* 1984;25:483–95. 10.1086/203165

[48] Lieberman DE, Raichlen DA, Pontzer H et al. The human gluteus maximus and its role in running. *J Exp Biol* 2006;209:2143–55. 10.1242/jeb.02255 16709916

[49] Liebenberg L . Persistence hunting by modern hunter-gatherers. *Curr Anthropol* 2006;47:1017–26. 10.1086/508695

[50] Pennington CW . The Tarahumar of Mexico: Their Environment and Material Culture. Utah: University of Utah Press, 1963. [cited 2026 Jun 3]. https://ehrafworldcultures.yale.edu/cultures/nu33/documents/012

[51] Zahavi A . Mate selection—a selection for a handicap. *J Theor Biol* 1975;53:205–14. 10.1016/0022-5193(75)90111-3 1195756

[52] Hill K, Kaplan H, Hawkes K. On why male foragers hunt and share food. *Curr Anthropol* 1993;34:701–10. 10.1086/204213

[53] Kaplan H, Hill K. Hunting ability and reproductive success among male ache foragers: preliminary results. *Curr Anthropol* 1985;26:131–3. [cited 2026 Jun 3] https://www.jstor.org/stable/2743006. 10.1086/203235

[54] Longman DP . Fitness benefits of costly signalling. In: Shackelford TK, Weekes-Shackelford VA (eds.), *Encyclopedia of Evolutionary Psychological Science*, pp. 3153–8. Cham: Springer, 2021 [cited 2026 Jun 3] https://link.springer.com/rwe/, 10.1007/978-3-319-19650-3_3493

[55] Longman D, Wells JCK, Stock JT. Can persistence hunting signal male quality? A test considering digit ratio in endurance athletes. *PLoS One* 2015;10:e0121560. 10.1371/journal.pone.0121560 PMC4390232 25853679

[56] Marlowe FW . Mate preferences among Hadza hunter-gatherers. *Hum Nat* 2004;15:365–76. 10.1007/s12110-004-1014-8 26189412

[57] Longman DP, Todorova Y, Bailey SJ et al. Outpaced by industry: industrial environments reduce endurance, with implications for evolutionary fitness. *Am J Biol Anthropol* 2026;190:e70270. 10.1002/ajpa.70270 42159240

[58] Lieberman DE, Bramble DM, Raichlen DA et al. Brains, brawn, and the evolution of human endurance running capabilities. In: Grine FE, Fleagle JG, Leakey RE (eds.), *The First Humans – Origin and Early Evolution of the Genus Homo*, pp. 77–92. Dordrecht: Springer Netherlands, 2009 [cited 2025 Oct 6] 10.1007/978-1-4020-9980-9_8.

[59] Borg G . Borg’s Perceived Exertion and Pain Scales, Vol. viii. Champaign, IL, US: Human Kinetics, 1998. 104.

[60] Hutchinson MJ, Kouwijzer I, de Groot S et al. Comparison of two Borg exertion scales for monitoring exercise intensity in able-bodied participants, and those with paraplegia and tetraplegia. *Spinal Cord* 2021;59:1162–9. 10.1038/s41393-021-00642-4 PMC8560635 34040150

[61] MacIntosh BR, Murias JM, Keir DA et al. What is moderate to vigorous exercise intensity? *Front Physiol* 2021;12:682233. 10.3389/fphys.2021.682233 PMC8493117 34630133

[62] Grove JR, Prapavessis H. Preliminary evidence for the reliability and validity of an abbreviated profile of mood states. *Int J Sport Psychol* 1992;23:93–109.

[63] WHO . Global physical activity questionnaire (GPAQ). 2021 [cited 2025 Jan 16]. https://www.who.int/publications/m/item/global-physical-activity-questionnaire

[64] WHO . WHO guidelines on physical activity and sedentary behaviour. 2020 [cited 2025 Dec 12]. https://www.who.int/publications/i/item/9789240015128 33369898

[65] Vermunicht P, Makayed K, Meysman P et al. Validation of Polar H10 chest strap and Fitbit inspire 2 tracker for measuring continuous heart rate in cardiac patients: impact of artefact removal algorithm. *EP Eur* 2023;25:euad122.550. 10.1093/europace/euad122.550

[66] Kendzierski D, DeCarlo KJ. Physical activity enjoyment scale: two validation studies. *J Sport Exerc Psychol* 1991;13:50–64. 10.1123/jsep.13.1.50

[67] Focht BC . Brief walks in outdoor and laboratory environments: effects on affective responses, enjoyment, and intentions to walk for exercise. *Res Q Exerc Sport* 2009;80:611–20. 10.1080/02701367.2009.10599600 19791648

[68] Bray SR, Millen JA, Eidsness J et al. The effects of leadership style and exercise program choreography on enjoyment and intentions to exercise. *Psychol Sport Exerc* 2005;6:415–25. 10.1016/j.psychsport.2004.07.003

[69] Fox LD, Rejeski WJ, Gauvin L. Effects of leadership style and group dynamics on enjoyment of physical activity. *Am J Health Promot* 2000;14:277–83. 10.4278/0890-1171-14.5.277 11009853

[70] Li Q . Effect of forest bathing trips on human immune function. *Environ Health Prev Med* 2010;15:9–17. 10.1007/s12199-008-0068-3 PMC2793341 19568839

[71] Armour M, Parry KA, Steel K et al. Australian female athlete perceptions of the challenges associated with training and competing when menstrual symptoms are present. *Int J Sports Sci Coach* 2020;15:316–23. 10.1177/1747954120916073

[72] Allen M, Poggiali D, Whitaker K et al. Raincloud plots: a multi-platform tool for robust data visualization. *Wellcome Open Res* 2021;4:63. 10.12688/wellcomeopenres.15191.2 PMC6480976 31069261

[73] Jensen K, Johansen L, Karkkainen OP. Economy in track runners and orienteers during path and terrain running. *J Sports Sci* 1999;17:945–50. 10.1080/026404199365335 10622354

[74] Voloshina AS, Ferris DP. Biomechanics and energetics of running on uneven terrain. *J Exp Biol* 2015;218:711–9. 10.1242/jeb.106518 25617451

[75] Gravina-Cognetti F, Chaverri D, Planas A et al. Mechanical running power and energy expenditure in uphill and downhill running. *Sports.* 2025;13:294. 10.3390/sports13090294 PMC12473670 41003600

[76] Lemire M, Falbriard M, Aminian K et al. Level, uphill, and downhill running economy values are correlated except on steep slopes. *Front Physiol* 2021;12:697315. 10.3389/fphys.2021.697315 PMC8281813 34276417

[77] Vernillo G, Giandolini M, Edwards WB et al. Biomechanics and physiology of uphill and downhill running. *Sports Med* 2017;47:615–29. 10.1007/s40279-016-0605-y 27501719

[78] Cavanagh PR, Lafortune MA. Ground reaction forces in distance running. *J Biomech* 1980;13:397–406. 10.1016/0021-9290(80)90033-0 7400169

[79] Cutrufello PT, Rundell KW, Smoliga JM et al. Inhaled whole exhaust and its effect on exercise performance and vascular function. *Inhal Toxicol* 2011;23:658–67. 10.3109/08958378.2011.604106 21867399

[80] Chen B, Kan H. Air pollution and population health: a global challenge. *Environ Health Prev Med* 2008;13:94–101. 10.1007/s12199-007-0018-5 PMC2698272 19568887

[81] Marr LC, Ely MR. Effect of air pollution on marathon running performance. *Med Sci Sports Exerc* 2010;42:585–91. 10.1249/MSS.0b013e3181b84a85 19952812

[82] Cusick M, Rowland ST, DeFelice N. Impact of air pollution on running performance. *Sci Rep* 2023;13:1832. 10.1038/s41598-023-28802-x PMC9892497 36725956

[83] Gao Y, Chan EYY, Zhu Y et al. Adverse effect of outdoor air pollution on cardiorespiratory fitness in Chinese children. *Atmos Environ* 2013;64:10–7. 10.1016/j.atmosenv.2012.09.063

[84] Yu ITS, Wong TW, Liu HJ. Impact of air pollution on cardiopulmonary fitness in schoolchildren. *J Occup Environ Med* 2004;46:946–52. 10.1097/01.jom.0000139871.50524.f6 15354060

[85] Adams WC, Schelegle ES. Ozone and high ventilation effects on pulmonary function and endurance performance. *J Appl Physiol* 1983;55:805–12. 10.1152/jappl.1983.55.3.805 6629918

[86] Mullins JT . Ambient air pollution and human performance: contemporaneous and acclimatization effects of ozone exposure on athletic performance. *Health Econ* 2018;27:1189–200. 10.1002/hec.3667 29737586

[87] UK National Atmospheric Emissions Inventory. [cited 2025 Apr 22]. https://naei.energysecurity.gov.uk/emissionsapp/

[88] Schraufnagel DE . The health effects of ultrafine particles. *Exp Mol Med* 2020;52:311–7. 10.1038/s12276-020-0403-3 PMC7156741 32203102

[89] Byrka K, Ryczko N. Positive effects of dancing in natural versus indoor settings: the mediating role of engagement in physical activity. *J Environ Psychol* 2018;57:25–33. 10.1016/j.jenvp.2018.06.002

[90] Reich AH, Queathem EJ. Setting, age, and intensity influence responses to exercise in young endurance runners. *Percept Mot Skills* 2020;127:533–54. 10.1177/0031512520903907 32036749

[91] Miller JR, Van Hooren B, Bishop C et al. A systematic review and meta-analysis of crossover studies comparing physiological, perceptual and performance measures between treadmill and Overground running. *Sports Med* 2019;49:763–82. 10.1007/s40279-019-01087-9 30847825

[92] Brand R, Ekkekakis P. Affective–reflective theory of physical inactivity and exercise. *Ger J Exerc Sport Res* 2018;48:48–58. 10.1007/s12662-017-0477-9

[93] Hagger MS, Chatzisarantis NLD. Integrating the theory of planned behaviour and self-determination theory in health behaviour: a meta-analysis. *Br J Health Psychol* 2009;14:275–302. 10.1348/135910708X373959 18926008

[94] Hagger MS . Habit and physical activity: theoretical advances, practical implications, and agenda for future research. *Psychol Sport Exerc* 2019;50 years of FEPSAC: Current and future directions to sport and exercise Psychology research;42:118–29. 10.1016/j.psychsport.2018.12.007

[95] Yang Z, Li J, Liu X. “Inconsistency between words and deeds”: a meta-analysis of the moderating and mediating mechanisms of bridging the exercise-intentional-behavior gap. *Front Psychol* 2025;16:1586176. 10.3389/fpsyg.2025.1586176 PMC12133765 40470022

[96] Ajzen I . The theory of planned behavior. Organ Behav hum Decis process. *Theories of Cognitive Self-Regulation* 1991;50:179–211. 10.1016/0749-5978(91)90020-T

[97] McEachan RRC, Conner M, Taylor NJ et al. Prospective prediction of health-related behaviours with the theory of planned behaviour: a meta-analysis. *Health Psychol Rev* 2011;5:97–144. 10.1080/17437199.2010.521684

[98] Dishman RK, Motl RW, Saunders R et al. Enjoyment mediates effects of a school-based physical-activity intervention. *Med Sci Sports Exerc* 2005;37:478–87. 10.1249/01.MSS.0000155391.62733.A7 15741848

[99] Dacey M, Baltzell A, Zaichkowsky L. Older adults’ intrinsic and extrinsic motivation toward physical activity. *Am J Health Behav* 2008;32:570–82. 10.5555/ajhb.2008.32.6.570 18442337

[100] Kohlstedt S, Weissbrod C, Colangelo AM et al. Psychological factors influencing exercise adherence among females. *Sci Res* 2013;04:917–23. 10.4236/psych.2013.412132

[101] Wells JCK, Nesse RM, Sear R et al. Evolutionary public health: introducing the concept. *Lancet* 2017;390:500–9. 10.1016/S0140-6736(17)30572-X 28792412

