# Supplementary material for: Running wild: Forest running enhances performance, enjoyment, mood, and future exercise intentions
Source: Evol Med Public Health. 2026 Aug 27;14(1):1–14. doi: 10.1093/emph/eoag018 (PMC13623525; doi:10.1093/emph/eoag018)
Supplement: Supplementary file 1 [file emph_supplementary_information_eoag018.docx]

**Supplementary Information 1**

|  | **Male (*n* = 31)** |  | **Female (*n* = 16)** |  |
| --- | --- | --- | --- | --- |
|  | **Mean (*SD*)** | **Range** | **Mean (*SD*)** | **Range** |
| **Moderate PA (per week)** |  |  |  |  |
| Weekly PA (METS) | 2,100 (1,400) | 200-7,000 | 1,800 (600) | 480-2,500 |
| Weekly PA (mins) | 460 (320) | 40-1500 | 390 (130) | 110-560 |
| Participants completing 150’ moderate PA (600 METS) | 29/31 |  | 15/16 |  |
| Participants completing 300’ moderate PA (1,200 METS) | 21/31 |  | 13/16 |  |
| **Vigorous PA (per week)** |  |  |  |  |
| Weekly PA (METS) | 1,100 (1,200) | 0-4,800 | 600 (600) | 0-1,900 |
| Weekly PA (mins) | 140 (150) | 0-600 | 80 (80) | 0-240 |
| Participants completing 75’ vigorous PA (600 METS) | 18/31 |  | 8/16 |  |
| Participants completing 150’ vigorous PA (1,200 METS) | 13/31 |  | 3/16 |  |
|  |  |  |  |  |

**Table S1.** The habitual physical activity of the participant cohort, as assessed by the 16-item Global Physical Activity Questionnaire (GPAQ), developed by the World Health Organisation (WHO) ^[1–3]^.

**Supplementary Information 2**

**Industrial site**

The urban component of this study was conducted at Bishop Meadow Industrial Estate, on the northern outskirts of Loughborough (Leicestershire, UK). Bishop Meadow is a well‑established mixed light‑industrial and commercial estate, comprising warehouses, manufacturing and distribution facilities, industrial workshops and associated office suites. The estate features dense built infrastructure, including steel‑framed buildings, loading docks, large hardstanding surfaces (roads, parking lots, loading yards), and direct access to major road arteries (e.g., the A6 trunk road) and motorway networks (M1 via the A512), facilitating logistics and freight transport associated with the industrial uses.

Loughborough itself is a sizeable market and university town with a population of approximately 65,000, making it the second-largest settlement in Leicestershire. Home to the internationally recognised Loughborough University, the town combines academic and industrial functions, and benefits from strong transport links by road, rail and air via nearby East Midlands Airport. As such, Bishop Meadow Industrial Estate typifies an urban/industrial environment in a mid-sized English town.

**Forest site**

The forest component of this study was undertaken in an area of semi-natural woodland within ‘Outwoods’, Leicestershire. Outwoods is an ancient semi-natural woodland (i.e. continuously forested since at least 1600 and with ecological characteristics of native, natural habitats), part of the Beacon Hill, Hangingstone and Outwoods Site of Special Scientific Interest (SSSI). The woodland in the study area is classified as ‘W10’ (Quercus robur – Pteridium aquilinum – Rubus fruticosus woodland) under the National Vegetation Classification typology. This woodland type is widespread throughout lowland England and Wales. In the study area, species composition was characterized by extensive past tree felling, resulting in abundant Betula pendula (Silver Birch) and Sorbus aucuparia (Rowan/Mountain Ash). For further floral information about the local area, see the attached reference to another study which conducted a floral survey and measurements of airborne volatile organic compounds within the same woodland ^[4]^.

**Supplementary Information 3. Model statistics**

**Short-term PA intention**

| **Fixed Effect** | **B** | ***SE*** | **df** | ***t*** | ***p*** | **95%CI** |
| --- | --- | --- | --- | --- | --- | --- |
| Intercept (Urban) | 34.1 | 4.0 | 30.8 | 8.564 | <0.001 | [26.0, 42.2] |
| Environment (Forest vs Urban) | 46.8 | 4.4 | 19.4 | 10.747 | <0.001 | [37.7, 55.9] |
| Order (Forest-Urban vs Urban-Forest) | -6.9 | 4.7 | 35.9 | -1.464 | 0.152 | [-16.5, 2.7] |
| **Random Effect** | **Variance** | ***SD*** |  |  |  |  |
| Participant ID (Intercept) | 41.8 | 6.5 |  |  |  |  |
| Group ID (Intercept) | 11.2 | 3.3 |  |  |  |  |
| Residual | 416.4 | 20.4 |  |  |  |  |

**Table S2. Results** **of linear mixed-effects models examining the effects of environment on short-term PA intention.** Linear mixed-effects model includ0ing fixed effects of Environment (forest, city) and Order (Forest-Urban vs Urban-Forest), with random intercepts for participant ID and Group ID. The intercept represents the estimated mean short-term PA intention at the reference condition (Environment = Urban; Order = Urban-Forest). Positive coefficients indicate a higher short-term PA intention relative to the reference category. p values correspond to tests of fixed effects. Random-effects estimates represent the variance and standard deviation of participant-level intercepts and residual error. The full model (fixed and random effects) explained 60% of the total variance in short-term PA intention (conditional R² = 0.597).

**Long-term PA intention**

| **Fixed Effect** | **B** | ***SE*** | **df** | ***t*** | ***p*** | **95%CI** |
| --- | --- | --- | --- | --- | --- | --- |
| Intercept (Urban) | 26.0 | 4.5 | 91 | 5.833 | <0.001 | [17.2, 34.9] |
| Environment (Forest vs Urban) | 50.4 | 4.9 | 91 | 10.393 | <0.001 | [40.8, 60.1] |
| Order (Forest-Urban vs Urban-Forest) | -10.0 | 5.4 | 91 | -1.871 | 0.065 | [-20.6, 0.6] |
| **Random Effect** | **Variance** | ***SD*** |  |  |  |  |
| Participant ID (Intercept) | 59.5 | 7.7 |  |  |  |  |
| Group ID (Intercept) | 0.0 | 0.0 |  |  |  |  |
| Residual | 553.7 | 23.5 |  |  |  |  |

**Table S3. Results of linear mixed-effects models examining the effects of environment on long-term PA intention.** Linear mixed-effects model including fixed effects of Environment (forest, city) and Order (Forest-Urban vs Urban-Forest), with random intercepts for participant ID and group ID. The intercept represents the estimated mean long-term PA intention at the reference condition (Environment = Urban; Order = Urban-Forest). Positive coefficients indicate a higher long-term PA intention relative to the reference category. p values correspond to tests of fixed effects. Random-effects estimates represent the variance and standard deviation of participant-level intercepts and residual error. The full model (fixed and random effects) explained 57% of the total variance in long-term PA intention (conditional R² = 0.568).

**Enjoyment**

| **Fixed Effect** | **B** | ***SE*** | **df** | ***t*** | ***p*** | **95%CI** |
| --- | --- | --- | --- | --- | --- | --- |
| Intercept (Urban) | 74.1 | 3.5 | 41.7 | 21.073 | <0.001 | [67.0, 81.2] |
| Environment (Forest vs Urban) | 24.8 | 3.6 | 32.5 | 6.986 | <0.001 | [17.6, 32.1] |
| Order (Forest-Urban vs Urban-Forest) | -0.3 | 4.2 | 40.8 | -0.075 | 0.940 | [8.7, 8.1] |
| **Random Effect** | **Variance** | ***SD*** |  |  |  |  |
| Participant ID (Intercept) | 59.1 | 7.7 |  |  |  |  |
| Group ID (Intercept) | 55.7 | 7.5 |  |  |  |  |
| Residual | 170.0 | 13.0 |  |  |  |  |

**Table S4. Results of linear mixed-effects models examining the effects of environment on enjoyment.** Linear mixed-effects model including fixed effects of Environment (forest, city) and Order (Forest-Urban vs Urban-Forest), with random intercepts for participant ID and group ID. The intercept represents the estimated mean enjoyment at the reference condition (Environment = Urban; Order = Urban-Forest). Positive coefficients indicate higher enjoyment relative to the reference category. p values correspond to tests of fixed effects. Random-effects estimates represent the variance and standard deviation of participant-level intercepts and residual error. The full model (fixed and random effects) explained 61% of the total variance in enjoyment (conditional R² = 0.614).

**Total mood disturbance (TMD)**

| **Fixed Effect** | **F** | **Numerator df** | **Denominator df** | ***p*** |
| --- | --- | --- | --- | --- |
| Intercept | 2070.744 | 1 | 183 | <0.001 |
| Environment | 11.332 | 1 | 183 | <0.001 |
| Time | 5.670 | 1 | 183 | 0.018 |
| Environment*Time | 24.594 | 1 | 183 | <0.001 |
| Order | 1.707 | 1 | 183 | 0.193 |
| **Random Effect** | **Variance** | ***SD*** |  |  |
| Participant ID (Intercept) | 153.0 | 12.4 |  |  |
| Group ID (Intercept) | 0.0 | 0.0 |  |  |
| Residual | 78.9 | 8.9 |  |  |

**Table S5. Results of linear mixed-effects models examining the effects of environment and time on total mood disturbance (TMD).** Linear mixed-effects model including fixed effects of Environment, Time (pre-/post-run), Environment × Time and Order (Forest-Urban vs Urban-Forest), with random intercepts for participant ID and group ID. Lower TMD scores indicate better mood. F values and p values correspond to Type III tests of fixed effects. Random-effects estimates represent the variance and standard deviation of participant-level intercepts and residual error. The full model (fixed and random effects) explained 69% of the total variance in TMD (conditional R² = 0.691)."

**Running performance (distance covered)**

| **Fixed Effect** | **B** | ***SE*** | **df** | ***t*** | ***p*** | **95%CI** |
| --- | --- | --- | --- | --- | --- | --- |
| Intercept (Urban) | 4,055.7 | 167.7 | 47.2 | 24.187 | <0.001 | [3,718.4, 4,393.0] |
| Environment (Forest vs Urban) | 182.1 | 81.0 | 19.9 | 2.248 | 0.036 | [13.0, 351.2] |
| Order (Forest-Urban vs Urban-Forest) | -240.4 | 231.6 | 44.7 | -1.038 | 0.305 | [-706.9, 226.0] |
| **Random Effect** | **Variance** | ***SD*** |  |  |  |  |
| Participant ID (Intercept) | 553,646.8 | 743.4 |  |  |  |  |
| Group ID (Intercept) | 18,769.2 | 136.9 |  |  |  |  |
| Residual | 108,677.8 | 329.6 |  |  |  |  |

**Table S6. Results of linear mixed-effects models examining the effects of environment on running performance (distance covered).** Linear mixed-effects model including fixed effects of Environment (forest, urban) and Order (Forest-Urban vs Urban-Forest), with random intercepts for participant ID and group ID. The intercept represents the estimated mean running distance at the reference condition (Environment = Urban; Order = Forest-Urban). Positive coefficients indicate greater running performance relative to the reference category. p values correspond to tests of fixed effects. Random-effects estimates represent the variance and standard deviation of participant-level intercepts and residual error. The full model (fixed and random effects) explained 85% of the total variance in running performance (conditional R² = 0.846).

**Rating of perceived exertion (RPE)**

| **Fixed Effect** | **B** | ***SE*** | **df** | ***t*** | ***p*** | **95%CI** |
| --- | --- | --- | --- | --- | --- | --- |
| Intercept (Urban) | 4.2 | 0.2 | 91 | 27.629 | <0.001 | [3.9, 4.5] |
| Environment (Forest vs Urban) | -0.1 | 0.1 | 91 | -0.628 | 0.531 | [-0.4, 0.2] |
| Order (Forest-Urban vs Urban-Forest) | 0.3 | 0.2 | 91 | 1.371 | 0.174 | [-0.1, 0.7] |
| **Random Effect** | **Variance** | ***SD*** |  |  |  |  |
| Participant ID (Intercept) | 0.2 | 0.5 |  |  |  |  |
| Group ID (Intercept) | 00 | 0.0 |  |  |  |  |
| Residual | 0.4 | 0.7 |  |  |  |  |

**Table S7.** Results of linear mixed-effects models examining the effects of environment on rating of perceived exertion (RPE). Linear mixed-effects model including fixed effects of Environment (forest, urban) and Order (Forest-Urban vs Urban-Forest), with random intercepts for participant ID and group ID. The intercept represents the estimated mean RPE at the reference condition (Environment = Urban; Order = Forest-Urban). p values correspond to tests of fixed effects. Random-effects estimates represent the variance and standard deviation of participant-level intercepts and residual error. The full model (fixed and random effects) explained 37% of the total variance in RPE (conditional R² = 0.374).

**Running average heart rate (HR)**

| **Fixed Effect** | **B** | ***SE*** | **df** | ***t*** | ***p*** | **95%CI** |
| --- | --- | --- | --- | --- | --- | --- |
| Intercept (Urban) | 159.8 | 2.8 | 73 | 57.683 | <0.001 | [154.2, 165.3] |
| Environment (Forest vs Urban) | -0.4 | 1.9 | 73 | -0.225 | 0.823 | [-4.3, 3.4] |
| Order (Forest-Urban vs Urban-Forest) | -3.3 | 3.7 | 73 | -0.897 | 0.373 | [-10.6, 4.0] |
| **Random Effect** | **Variance** | ***SD*** |  |  |  |  |
| Participant ID (Intercept) | 93.5 | 9.7 |  |  |  |  |
| Group ID (Intercept) | 0.0 | 0.0 |  |  |  |  |
| Residual | 69.7 | 8.4 |  |  |  |  |

**Table S8. Results of linear mixed-effects models examining the effects of environment on average heart rate (HR) during running.** Linear mixed-effects model including fixed effects of Environment (forest, urban) and Order (Forest-Urban vs Urban-Forest), with random intercepts for participant ID and group ID. The intercept represents the estimated mean HR at the reference condition (Environment = Urban; Order = Forest-Urban). p values correspond to tests of fixed effects. Random-effects estimates represent the variance and standard deviation of participant-level intercepts and residual error. The full model (fixed and random effects) explained 58% of the total variance in HR (conditional R² = 0.580).

**Supplementary Information 4**

| Variables | Forest Male Mean±*SD*  (*n* = 31) | Urban Male Mean±*SD*  (*n* = 31) | Forest Female Mean±*SD*  (*n* = 16) | Urban Female Mean±*SD*  (*n* = 16) |
| --- | --- | --- | --- | --- |
| Running distance (m) | 4,313±926.3 | 4,081±732.9 | 3,733±710.9 | 3,681±704.1 |
| Rating of perceived exertion | 4.2±0.7 | 4.3±0.7 | 4.4±1.0 | 4.6±1.0 |
| Heart rate (bpm) | 159±13.7* | 159±12.3* | 154±12.8** | 155±11.2** |
| Short-term intention (%) | 79.3±15.0 | 38±27.8 | 74.4±17.7 | 15.9±15.7 |
| Long-term intention (%) | 72.0±24.3 | 27.9±29.9 | 70.8±23.2 | 7.94±9.4 |
| Enjoyment | 99.8±13.3 | 76.0±19.4 | 97.6±11.7 | 68.9±20.5 |
| Mood | 88.0±15.9  🡪78.8±9.8 | 84.7±11.9  🡪89.7±16.6 | 93.2±19.8  🡪83.1±10.9 | 93.6±17.5  🡪93.6±20.7 |
| **n* = 26, ***n* = 12 |  |  |  |  |

**Table S9. Unadjusted variable means, split by environment and sex.** Overview of unadjusted male and female means with standard deviation, split by forest and urban environments.

**References**

1.	WHO. Global physical activity questionnaire (GPAQ) [Internet]. 2021 [cited 2025 Jan 16]. Available from: https://www.who.int/publications/m/item/global-physical-activity-questionnaire

2.	Cleland CL, Hunter RF, Kee F, Cupples ME, Sallis JF, Tully MA. Validity of the Global Physical Activity Questionnaire (GPAQ) in assessing levels and change in moderate-vigorous physical activity and sedentary behaviour. BMC Public Health. 2014;14(1):1255. doi:10.1186/1471-2458-14-1255

3.	Armstrong T, Bull F. Development of the World Health Organization Global Physical Activity Questionnaire (GPAQ). J Public Health. 2006;14(2):66–70. doi:10.1007/s10389-006-0024-x

4.	Longman DP, Todorova Y, Bailey SJ, Bishop NC, Davidson M, Drewer J, et al. Outpaced by Industry: Industrial Environments Reduce Endurance, With Implications for Evolutionary Fitness. Am J Biol Anthropol. 2026;190(1):e70270. doi:10.1002/ajpa.70270
